# Recruitment and representativeness of blood donors in the INTERVAL randomised trial assessing varying inter-donation intervals

**DOI:** 10.1186/s13063-016-1579-7

**Published:** 2016-09-20

**Authors:** Carmel Moore, Thomas Bolton, Matthew Walker, Stephen Kaptoge, David Allen, Michael Daynes, Susan Mehenny, Jennifer Sambrook, Nicholas A. Watkins, Gail Miflin, Emanuele Di Angelantonio, Willem H. Ouwehand, David J. Roberts, John Danesh, Simon G. Thompson

**Affiliations:** 1 Department of Public Health and Primary Care, University of Cambridge, Strangeways Research Laboratory, Worts Causeway, Cambridge, CB1 8RN UK; 2 NHS Blood and Transplant – Oxford Centre, Level 2, John Radcliffe Hospital, Headley Way, Oxford, OX3 9BQ UK; 3 NHS Blood and Transplant, Northway, Filton, Bristol, BS34 7QH UK; 4 NHS Blood and Transplant, Longley Lane, Sheffield, S5 7JN UK; 5 Department of Haematology, University of Cambridge, Cambridge Biomedical Campus, Long Road, Cambridge, CB2 OPT UK; 6 NHS Blood and Transplant, Cambridge Biomedical Campus, Long Road, Cambridge, CB2 OPT UK; 7 NHS Blood and Transplant, Reeds Crescent, Watford, Herts WD24 4QN UK; 8 NIHR Blood and Transplant Research Unit in Donor Health and Genomics, Strangeways Research Laboratory, Worts Causeway, Cambridge, CB1 8RN UK; 9 Wellcome Trust Sanger Institute, Wellcome Genome Campus, Hinxton, Cambridge, CB10 1SA UK; 10 Radcliffe Department of Medicine, University of Oxford, John Radcliffe Hospital, Headley Way, Oxford, OX3 9DU UK; 11 Department of Public Health and Primary Care, The INTERVAL Trial Coordinating Centre, University of Cambridge, Cambridge, CB1 8RN UK

**Keywords:** Randomised trial, Recruitment, Representativeness, Generalisability, Blood donors, Blood donation

## Abstract

**Background:**

The interpretation of trial results can be helped by understanding how generalisable they are to the target population for which inferences are intended. INTERVAL, a large pragmatic randomised trial of blood donors in England, is assessing the effectiveness and safety of reducing inter-donation intervals. The trial recruited mainly from the blood service’s static centres, which collect only about 10 % of whole-blood donations. Hence, the extent to which the trial’s participants are representative of the general blood donor population is uncertain. We compare these groups in detail.

**Methods:**

We present the CONSORT flowchart from participant invitation to randomisation in INTERVAL. We compare the characteristics of those eligible and consenting to participate in INTERVAL with the general donor population, using the national blood supply ’PULSE’ database for the period of recruitment. We compare the characteristics of specific groups of trial participants recruited from different sources, as well as those who were randomised versus those not randomised.

**Results:**

From a total of 540,459 invitations, 48,725 donors were eligible and consented to participate in INTERVAL. The proportion of such donors varied from 1–22 % depending on the source of recruitment. The characteristics of those consenting were similar to those of the general population of 1.3 million donors in terms of ethnicity, blood group distribution and recent deferral rates from blood donation due to low haemoglobin. However, INTERVAL participants included more men (50 % versus 44 %), were slightly older (mean age 43.1 versus 42.3 years), included fewer new donors (3 % versus 22 %) and had given more donations over the previous 2 years (mean 3.3 versus 2.2) than the general donor population. Of the consenting participants, 45,263 (93 %) donors were randomised. Compared to those not randomised, the randomised donors showed qualitatively similar differences to those described above.

**Conclusions:**

There was broad similarity of participants in INTERVAL with the general blood donor population of England, notwithstanding some differences in age, sex and donation history. Any heterogeneity of the trial’s results according to these characteristics will need to be studied to ensure its generalisability to the general donor population.

**Trial registration:**

Current Controlled Trials ISRCTN24760606. Registered on 25 January 2012.

**Electronic supplementary material:**

The online version of this article (doi:10.1186/s13063-016-1579-7) contains supplementary material, which is available to authorized users.

## Background

One of the key roles of the National Health Service Blood and Transplant (NHSBT) is to provide an efficient supply of blood and blood components to hospitals in England and North Wales. The INTERVAL trial is a parallel group, pragmatic, individually randomised controlled trial (RCT) which aimed to recruit approximately 50,000 blood donors registered with NHSBT [1]. INTERVAL seeks to address an important question for NHSBT and potentially blood services worldwide: What is the optimum frequency of whole-blood donation to enhance blood supplies and maintain donors’ health? The trial is sufficiently powered to provide evidence on whether donation intervals should be tailored to donor subgroups who are more or less susceptible to iron deficiency [2]. The trial’s objectives are driven by the need to meet the dual potential challenge of increases in demand for blood as the population ages, and difficulties in attracting new blood donors [3–5].

Giving a unit (about 0.5 litres) of whole blood is the most common type of blood donation and differs from other donations involving the collection of specific blood components such as red cells, platelets or plasma. In current NHSBT practice, male and female whole-blood donors can give blood as frequently as every 12 and 16 weeks, respectively. During their 2-year involvement in the INTERVAL trial, participants were randomised to give blood at either these standard donation intervals or more often, specifically every 12, 10 or 8 weeks for men and 16, 14 or 12 weeks for women. The trial’s primary outcome is the number of whole-blood units donated over 2 years, and the key secondary outcome is the Short Form Survey-36 composite measure of physical well-being at 2 years [6]; the trial’s statistical analysis plan is provided in Additional file 1. A criticism of research studies (especially RCTs) is a lack of consideration for external validity and whether the findings are generalisable to a wider setting than that in which they were initially tested [7]. Therefore, the extent to which the trial’s main findings (due to be reported in 2017) are applicable to future donation strategies will be influenced by how representative the INTERVAL participants are of the general donor population.

Anyone between the ages of 17 and 65 years can register with NHSBT as a blood donor; once registered a donor may continue donating up to and beyond 65 years old if they meet all usual criteria for giving blood. In 2013, more than 1.7 million whole-blood donations were made by almost 1 million donors at more than 23,000 donation venues across England and North Wales [8]. Approximately 90 % of these donations were made at community venues or ‘mobile sessions’ (e.g. village halls, local schools). The remaining 10 % were made at the 25 premises run by NHSBT as ‘static’ donor centres across England. The relatively small proportion of whole-blood collections at the static donor centres reflects the focus, at that time, on the collection of platelet donations at these sites.

In this paper we describe recruitment to INTERVAL and investigate how representative the trial participants were of the general donor population.

## Methods

### Selection and recruitment of participants

Recruitment of donors to the INTERVAL trial took place between June 2012 and June 2014. Donors were eligible to join the trial if they were aged 18 years or older (to meet ethical requirements, with the normal lower age limit for donation being 17 years old), fulfilled all routine criteria for blood donation, were willing to be randomised to any of the trial’s intervention groups and had an email address and access to the Internet (since the trial collects data mainly via remote and web-based methods). In addition, donors had to be willing to give blood at one of the 25 static donor centres; this condition was necessary to overcome NHSBT information technology safeguards to prevent donors giving at less than standard donation intervals.

Invitations were sent to specific groups (or ‘sources’) of donors during the recruitment period (Table 1). Initially postal invitations were sent to donors registered at one of the 25 static donor centres. However, in order to meet the trial’s recruitment target, we broadened the pool of potential participants by extending postal or email invitations to mobile session donors thought to be most likely to join the trial (and transfer to a static donor centre); email was preferred for large groups in order to reduce costs. In particular, we approached mobile session donors either because they had previously agreed to give platelet donations at static centres or because of the donor’s proximity to a static donor centre (judged by the location of their usual mobile session or NHSBT correspondence address). In addition, donors arriving at a static donor centre who had not received an invitation could also be informed about INTERVAL. Donors expressing an interest in the trial, and able to donate following NHSBT’s routine screening, were asked to complete the trial consent form prior to giving their usual donation [1]. Donors who were temporarily unable to donate (for reasons such as failure to meet haemoglobin thresholds, recent overseas travel) had the opportunity to join the trial at their next donation visit, as long as the visit occurred within the recruitment period.

**Table 1:** Description of recruitment sources for the INTERVAL trial

Recruitment source	Source description	Invitation period	Invitation type
Centre	Donors registered at a static donor centre	Jun 2012 – Jan 2013	Letter
Mobile (platelet)	Mobile session donors who had previously registered an interest in giving platelets at a static donor centre	Jan 2013 – Oct 2013	Letter
Mobile (10-mile)	Donors giving blood at a mobile session within 10 miles of a static donor centre	Jan 2013 – Oct 2013	Letter or email
Mobile (30-mile)	Donors with a correspondence address within 30 miles (but typically 20 miles) of a static donor centre	Jan 2013 – Oct 2013	Email
No invitation	Donors giving blood at a static donor centre who expressed an interest in joining the trial but who did not receive a previous invitation	Jun 2012 – Jun 2014	Not applicable

### Data collection

Data used to populate the CONSORT flowchart were derived from NHSBT’s national blood supply database (PULSE) and the INTERVAL trial’s research database. PULSE covers all aspects of donor and donation management: procedure codes are added to donors’ records according to the type of donations given (e.g. platelet, whole-blood), and communication codes are used to record types of messages sent to donors. New communication codes were added to PULSE for donors invited to join INTERVAL from different sources (Table 1), along with procedure codes to enable tracking of INTERVAL participants [1]. Further communication codes were placed on the records of donors who, on attending a static donor centre during the recruitment period, expressed an interest in and/or enrolled in the study.

These enrolment codes served to activate the secure transfer of participants’ trial-relevant data from PULSE to the INTERVAL research database (held at the academic coordinating centre at the University of Cambridge). Receipt of these data then triggered an email from the academic coordinating centre requesting consenting participants to complete the INTERVAL baseline questionnaire. Only those consenting participants who responded to this initial online questionnaire were subsequently randomised into the trial [1].

The PULSE database was also used to create a bespoke dataset on all registered NHSBT blood donors to compare the characteristics of the INTERVAL cohort with the general donor population. This dataset consisted of entries for 3,362,757 donors, including INTERVAL participants (identified by an anonymous study number). Information retrieved included individual-level data on donors’ sex, age and ethnicity, ABO and Rhesus D blood group, NHSBT registration date, date of first attendance for donation (whether successful or not), date of first successful donation and detailed information on the outcomes of each attendance over the past 7 years. Geographic reference data, held on PULSE, were used to calculate distances between donors’ correspondence address and their nearest static donor centre.

### Data analysis

The INTERVAL cohort is defined as blood donors who completed a consent form and were eligible to join the study. The INTERVAL trial comprises participants who additionally completed the baseline questionnaire and were randomised.

In defining recruitment source, participants receiving an invitation following their enrolment were deemed not to have received a postal/email invitation. Where participants had received more than one postal/email invitation type during the recruitment period, the invitation immediately prior to their enrolment in the study was used to indicate their source of recruitment. For donors who did not join the study and were sent more than one invitation, the first one sent was taken as their invitation type.

**Fig. 1:**
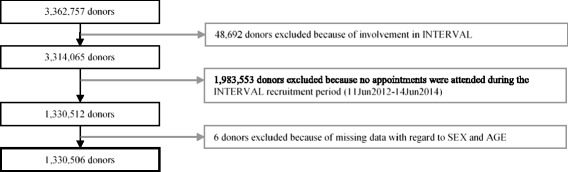
Flow diagram showing the selection of donors forming the NHSBT general donor population

The general donor population, used as a comparator, was defined as all donors registered with NHSBT who did not consent to INTERVAL but had attended an appointment during the INTERVAL recruitment period (11 June 2012 – 14 June 2014); see Fig. 1. To define donor characteristics it was necessary to fix an appropriate reference date. For INTERVAL participants this date corresponded to the date of their baseline attendance. For the general donor population, it was defined as participants’ date of attendance nearest to the mid-point of the INTERVAL recruitment period (12 June 2013). Age at baseline was calculated from the attendance date and recorded date of birth. Ethnicity and ABO/Rhesus D blood group were as recorded on NHSBT’s PULSE database.

The focus of the analysis is on the magnitude of differences between groups rather than their statistical significance. Given the very large numbers, even very small differences can have extreme *P* values. Hence, differences with 95 % confidence intervals (CI) are presented rather than *P* values.

## Results

### Participant recruitment

**Fig. 2:**
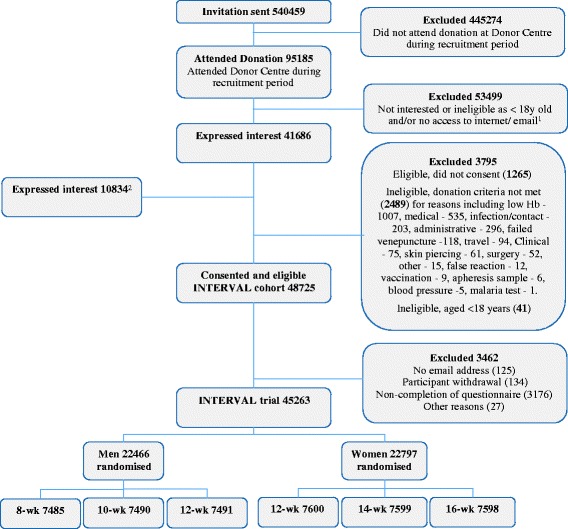
CONSORT flowchart of participants into the INTERVAL trial. ^1^On arrival at the donor centre, donation staff in the welcome area asked donors if they were interested in joining, were ≥18 years old and had access to email/Internet. If a donor responded ‘no’ to any of these questions, he/she was excluded. ^2^This group expressed an interest in joining the study when asked by donation staff in the welcome area, but had not previously received an invitation

Figure 2 summarises the status of participants at different stages of recruitment. Between June 2012 and October 2013, more than 500,000 blood donors were sent an invitation, just under 100,000 attended one of the 25 static donor centres during the recruitment period and of these 44 % expressed an interest in joining the trial and (on initial questioning) met the age and Internet/email criteria. There was a further group of approximately 11,000 donors who did not receive an invitation but who attended a recruitment centre for donation and responded positively when asked by donor-centre staff if they wished to enrol (the denominator for this group is unknown).

Data collected by donation staff using a pre-defined questionnaire indicated that the main reason given for not wanting to join the study was the time commitment required (41 % of donors who gave a reason). About 16 % of donors wanted greater flexibility with respect to donation appointments, 6 % were concerned about the donation frequency, 7 % were being evaluated as potential platelet donors, 1 % disagreed with the aims of the study and 29 % indicated that they had other reasons for not joining.

The majority of donors who expressed an initial interest in the trial then gave their consent to join the INTERVAL cohort (93 %) with only a small proportion who either did not meet the study/donation criteria or for other reasons decided not to enrol. Only those members of the INTERVAL cohort who completed the trial’s baseline questionnaire were subsequently randomised; as such the INTERVAL trial represents 93 % of the INTERVAL cohort (and 87 % of all those expressing an interest). The numbers of male and female randomised donors were similar, and there was an even split of donors into the gender-specific treatment allocations.

### Participation by sources of recruitment

The participation by subgroups of donors according to their sources of recruitment is shown in Table 2. The largest group of INTERVAL participants (31,945) came from the pool of donors who had attended a static donor centre prior to recruitment — this group showed the highest consent rate to the INTERVAL cohort (22.1 % of all invitations sent) and trial recruitment (20.4 %). In contrast, the recruitment rate was lower in mobile session donors who either donated within 10 miles (2.8 %) or had a correspondence address within 30 miles (1.3 %) of a static donor centre. As these sources were of a considerable size, however, their contribution to the INTERVAL cohort was not insubstantial (7,649 participants). The recruitment rate from the Mobile (platelet) group was higher than from the other mobile session recruitment sources, as expected due to their prior willingness to transfer to a static donor centre. However, as this group represented a small pool of donors, the overall number of recruited participants from this source was relatively small.

**Table 2:** Breakdown of CONSORT flowchart according to source of recruitment

	Invitations			Exclusions: Post-attendance and prior to consent	INTERVAL cohort and trial			
Recruitment source	Invited	Attended donation			INTERVAL cohort		Randomised	Not randomised
Centre	144,852	71,445	(49.3 %)	39,500	31,945	(22.1 %)	29,549	2396
Mobile (platelet)	21,585	2330	(10.8 %)	586	1744	(8.1 %)	1696	48
Mobile (10-mile)	190,081	15,886	(8.4 %)	10,648	5238	(2.8 %)	5009	229
Mobile (30-mile)	183,941	5524	(3.0 %)	3113	2411	(1.3 %)	2325	86
No invitation^a^	-	10,834	-	3447	7387	-	6684	703
Total	540,459	106,019		57,294	48,725		45,263	3462

^a^Walk-in donors without a previous invitation; no denominator available

### Comparison of INTERVAL donors to the general donor population

The INTERVAL cohort represented approximately 4 % of NHSBT’s 1.3 million general donor population. The characteristics of these groups are compared in Tables 3 and 4. Some notable differences were that the INTERVAL cohort included more men (50 % versus 44 %), were slightly older (mean age: 43.1 versus 42.3 years, with a lower proportion of 17–24 year olds: 13 % versus 18 %) and lived closer to a static donor centre (62 % versus 19 % within 0–4 miles, partly by design). In both samples the majority of individuals (91 %) reported their ethnicity as ‘white’ (Table 3); however, after excluding donors with missing values (Table 4), this proportion was slightly lower in the INTERVAL cohort. The distribution of blood groups was similar. By design the entire INTERVAL cohort attended a static donor centre at baseline. Several factors suggest that the INTERVAL cohort was a more long-standing and dedicated group of donors: they had a slightly longer history of donation with NHSBT (10.7 versus 8.6 years), a much lower proportion of new donors (3 % versus 22 %) and had given, on average, more whole-blood donations during the 2 years prior to baseline (mean 3.2 versus 2.1). The proportion of donors with a deferral over the previous 2 years (both for low haemoglobin and any other reason) appeared higher in the INTERVAL cohort. However, when this is expressed relative to the average number of whole-blood donations given, the deferral rate for low haemoglobin is similar to that in the general donor population (i.e. 2.1 % and 2.3 % per donation, respectively).

**Table 3:** Demographic characteristics of the NHSBT general donor population and of the INTERVAL cohort and trial

Demographics			INTERVAL cohort	
*n* (%) or mean (SD)	INTERVAL cohort	NHSBT general donor population	Randomised in INTERVAL trial	Not randomised
All donors	48,692 (100.0 %)^a^	1,330,506 (100.0 %)	45,235 (100.0 %)^a^	3457 (100.0 %)
Sex				
Male	24,212 (49.7 %)	586,372 (44.1 %)	22,456 (49.6 %)	1756 (50.8 %)
Female	24,480 (50.3 %)	744,134 (55.9 %)	22,779 (50.4 %)	1701 (49.2 %)
Age at baseline (years)	43.1 (14.3)	42.3 (15.0)	43.3 (14.2)	39.9 (14.7)
Age at baseline				
17–24	6424 (13.2 %)	234,367 (17.6 %)	5679 (12.6 %)	745 (21.6 %)
25–34	10,236 (21.0 %)	235,238 (17.7 %)	9468 (20.9 %)	768 (22.2 %)
35–44	9095 (18.7 %)	246,225 (18.5 %)	8500 (18.8 %)	595 (17.2 %)
45–-54	11,158 (22.9 %)	304,927 (22.9 %)	10,456 (23.1 %)	702 (20.3 %)
55–-64	8822 (18.1 %)	221,143 (16.6 %)	8362 (18.5 %)	460 (13.3 %)
65+	2957 (6.1 %)	88,606 (6.7 %)	2770 (6.1 %)	187 (5.4 %)
Ethnicity				
White	44,192 (90.8 %)	1,207,593 (90.8 %)	41,259 (91.2 %)	2933 (84.8 %)
Asian	1127 (2.3 %)	31,829 (2.4 %)	941 (2.1 %)	186 (5.4 %)
Black African	118 (0.2 %)	3852 (0.3 %)	105 (0.2 %)	13 (0.4 %)
Black Caribbean	307 (0.6 %)	4971 (0.4 %)	271 (0.6 %)	36 (1.0 %)
Black other	31 (0.1 %)	743 (0.1 %)	29 (0.1 %)	2 (0.1 %)
Chinese	164 (0.3 %)	3831 (0.3 %)	141 (0.3 %)	23 (0.7 %)
Mixed	679 (1.4 %)	14,838 (1.1 %)	606 (1.3 %)	73 (2.1 %)
Other	138 (0.3 %)	3558 (0.3 %)	122 (0.3 %)	16 (0.5 %)
Unknown	1936 (4.0 %)	59,291 (4.5 %)	1761 (3.9 %)	175 (5.1 %)
Blood group				
A RhD positive (A+)	14,737 (30.3 %)	397,277 (29.9 %)	13,768 (30.4 %)	969 (28.0 %)
A RhD negative (A-)	3744 (7.7 %)	97,450 (7.3 %)	3489 (7.7 %)	255 (7.4 %)
B RhD positive (B+)	4244 (8.7 %)	102,650 (7.7 %)	3877 (8.6 %)	367 (10.6 %)
B RhD negative (B-)	1070 (2.2 %)	27,317 (2.1 %)	993 (2.2 %)	77 (2.2 %)
O RhD positive (O+)	17,642 (36.2 %)	460,501 (34.6 %)	16,398 (36.3 %)	1244 (36.0 %)
O RhD negative (O-)	5542 (11.4 %)	138,349 (10.4 %)	5138 (11.4 %)	404 (11.7 %)
AB RhD positive (AB+)	1293 (2.7 %)	33,817 (2.5 %)	1199 (2.7 %)	94 (2.7 %)
AB RhD negative (AB-)	402 (0.8 %)	9550 (0.7 %)	368 (0.8 %)	34 (1.0 %)
Unknown	18 (0.0 %)	63,595 (4.8 %)	5 (0.0 %)	13 (0.4 %)
Distance to nearest static donor centre (miles)^b^				
0–4	30,405 (62.4 %)	252,068 (18.9 %)	27,971 (61.8 %)	2434 (70.4 %)
5–9	9801 (20.1 %)	245,652 (18.5 %)	9236 (20.4 %)	565 (16.3 %)
10–29	7203 (14.8 %)	584,850 (44.0 %)	6832 (15.1 %)	371 (10.7 %)
30–59	775 (1.6 %)	220,388 (16.6 %)	730 (1.6 %)	45 (1.3 %)
60+	98 (0.2 %)	18,045 (1.4 %)	93 (0.2 %)	5 (0.1 %)
Unknown	410 (0.8 %)	9503 (0.7 %)	373 (0.8 %)	37 (1.1 %)
Donor status at baseline^c^				
New	1392 (2.9 %)	288,653 (21.7 %)	1147 (2.5 %)	245 (7.1 %)
Occasional	7912 (16.2 %)	226,507 (17.0 %)	7053 (15.6 %)	859 (24.8 %)
More frequent	39,388 (80.9 %)	815,346 (61.3 %)	37,035 (81.9 %)	2353 (68.1 %)
Donations (over last 2 years prior to baseline)^d^				
All donations	3.28 (2.00)	2.24 (2.37)	3.33 (2.00)	2.61 (1.92)
Whole-blood donations	3.19 (1.81)	2.08 (1.87)	3.23 (1.80)	2.55 (1.80)
Other donations	0.10 (1.05)	0.16 (1.64)	0.10 (1.07)	0.06 (0.79)
Deferrals (during the 2 years prior to baseline)^e^				
Deferral for low haemoglobin	3308 (6.8 %)	63,071 (4.7 %)	3094 (6.8 %)	214 (6.2 %)
Any other deferral	14,628 (30.0 %)	327,747 (24.6 %)	13,632 (30.1 %)	996 (28.8 %)
Length of NHSBT donation history at baseline (years)^f^	10.7 (8.4)	8.58 (8.44)	10.9 (8.4)	8.47 (8.02)
Venue type attended at baseline^g^				
Static centre	48,692 (100.0 %)	99,724 (7.5 %)	45,235 (100.0 %)	3457 (100.0 %)
Mobile	0 (0.0 %)	1,230,782 (92.5 %)	0 (0.0 %)	0 (0.0 %)

^a^The characteristics of 33 donors in the INTERVAL cohort (of whom 28 are in the INTERVAL trial) could not be identified in the PULSE database due to merged donor records

^b^The correspondence address (used to calculate ’Distance to nearest static donor centre’ and derive ’Region’) was correct at the time the data were extracted from PULSE (Nov 2015). Historical correspondence addresses were not available in PULSE

^c^ ’New’ has been defined according to the classification used by NHSBT, i.e. an individual who has not previously provided a full donation is considered to be a new donor. ’Occasional’ and ’More frequent’ have been defined as less than or equal to two full donations in the last 5 years and more than two full donations in the last 5 years, respectively

^d^Including donations where volume equals ’Normal’, ’Overweight’ or ’Missing’ (not including donations where volume equals ’Empty’ or ’Underweight’)

^e^Deferrals during the 2 years prior to baseline were summarised as counts of people with a deferral for low haemoglobin levels and more generally deferral for reasons other than low haemoglobin

^f^Length of donation history with NHSBT at baseline was defined as the period of time between baseline and the minimum of date of registration, date of first attendance (whether successful or not) and date of first successful donation

^g^For venue type, some old venues that are no longer used do not have an associated PULSE venue code. In these cases, although venue type was missing, because the only venues that were no longer being used were mobile venues, we have assumed that the venue type was mobile

*SD*, Standard deviation

**Table 4:** Differences (95 % CIs): INTERVAL cohort versus NHSBT general donor population and INTERVAL randomised versus not randomised

Demographics				INTERVAL cohort		
mean or proportion (%)^a^	INTERVAL cohort	NHSBT general donor population	Difference (95 % CI)	Randomised	Not randomised	Difference (95 % CI)
Sex: Male (%)	49.7 %	44.1 %	5.7 % (5.2 %, 6.1 %)	49.6 %	50.8 %	-1.2 % (-2.9 %, 0.6 %)
Age at baseline (years)	43.1	42.3	0.73 (0.59, 0.87)	43.3	39.9	3.37 (2.88, 3.86)
Ethnicity: White (%)	94.5 %	95.0 %	-0.5 % (-0.7 %, -0.3 %)	94.9 %	89.4 %	5.5 % (4.5 %, 6.6 %)
Blood group: O (%)^b^	47.6 %	47.3 %	0.4 % (-0.1 %, 0.8 %)	47.6 %	47.9 %	-0.2 % (-2.0 %, 1.5 %)
Distance to nearest static donor centre (miles)	6.29	18.1	-11.8 (-12.0, -11.7)	6.37	5.24	1.12 (0.82, 1.43)
Number of all donations^c^	3.28	2.24	1.04 (1.02, 1.06)	3.33	2.61	0.73 (0.66, 0.80)
Deferral for low haemoglobin (%)^c^	6.8 %	4.7 %	2.1 % (1.8 %, 2.3 %)	6.8 %	6.2 %	0.6 % (-0.2 %, 1.5 %)
Any other deferral (%)^c^	30.0 %	24.6 %	5.4 % (5.0 %, 5.8 %)	30.1 %	28.8 %	1.3 % (-0.2 %, 2.9 %)
Length of NHSBT donation history at baseline (years)	10.7	8.58	2.15 (2.07, 2.22)	10.9	8.47	2.43 (2.14, 2.72)

^a^Those with missing values excluded

^b^Blood group O includes both RhD groups

^c^During the 2 years prior to baseline

### Geographical distribution of donors

**Fig. 3:**
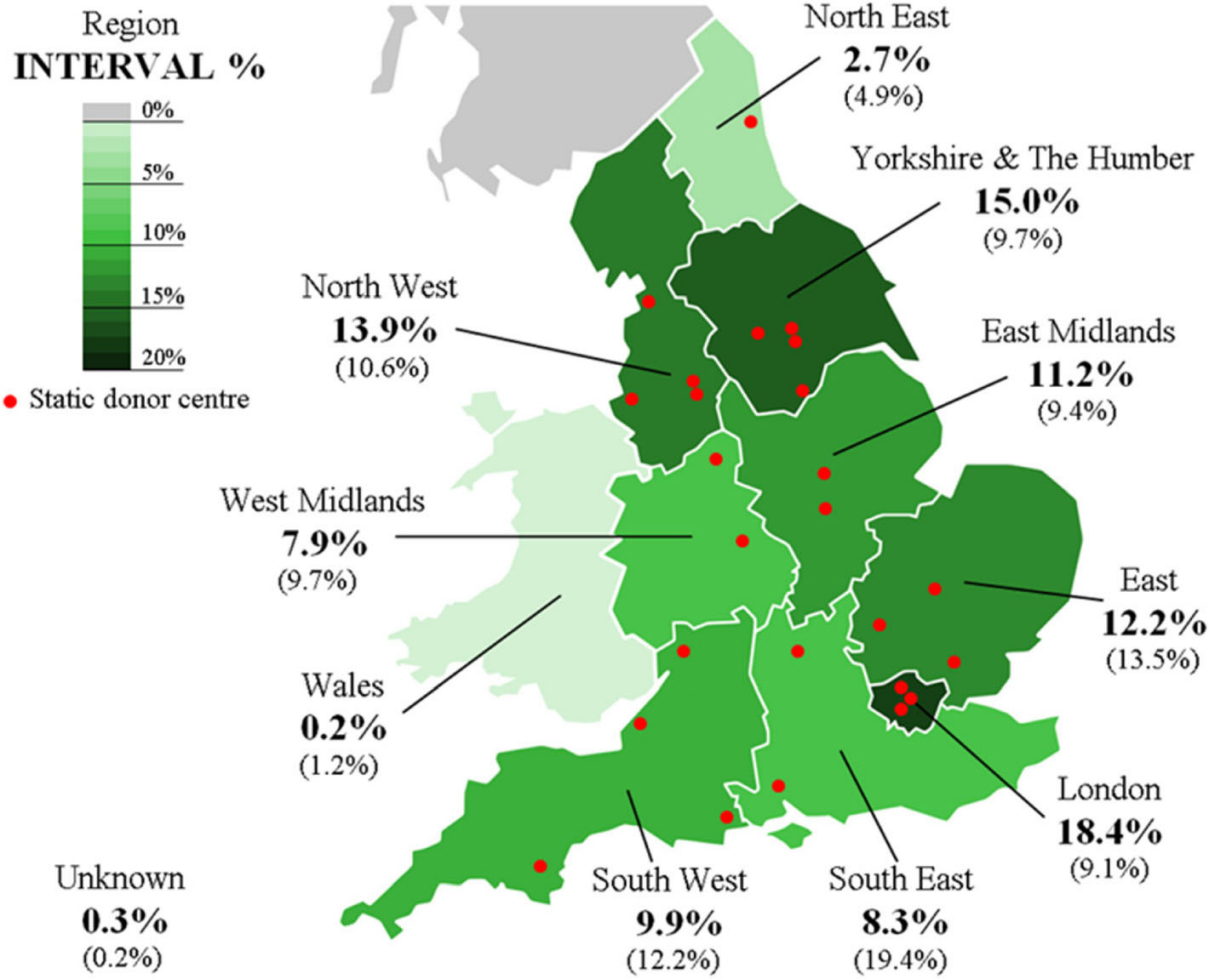
Geographical distribution of the INTERVAL cohort (NHSBT general donor population in brackets) by region

Figure 3 compares the geographical distribution of the INTERVAL cohort and NHSBT’s general donor population. For the most part, the proportion of INTERVAL participants in each of the ten regions was fairly similar to the general donor population. Regions in which the density of static donor centres is high (i.e. London, Yorkshire and the Humber and North West) showed the highest proportions of INTERVAL donors, which most notably exceeded those for the general donor population. The opposite was true in the South East and the North East, where there are very few static donor centres in relation to the size and density of the donor population, and in Wales, where there are none.

### Comparison of randomised versus non-randomised donors

Tables 3 and 4 also compare characteristics of participants finally randomised in the INTERVAL trial against those not randomised, primarily due to non-completion of the baseline questionnaire. Whereas the sex ratio was comparable, randomised participants were slightly older (mean age: 43 versus 40 years, with a lower proportion aged 17–24 years: 13 % versus 22 %), were more likely to be of white ethnicity (91 % versus 85 %), were more likely to have been a frequent donor (82 % versus 68 %) and had given a greater average number of donations in the 2 years prior to baseline (3.3 versus 2.6). The groups were similar in terms of the distribution of blood groups and proportion of deferrals for both low haemoglobin and any other reason. Surprisingly, the data do not suggest that members of the INTERVAL cohort who had a correspondence address at a greater distance from a static donor centre were less likely to be randomised.

### Comparison of donors by recruitment source

As shown in Table 5, the characteristics of the INTERVAL cohort participants from the Centre, Mobile (10-mile) and Mobile (30-mile) sources were generally similar (with some differences as expected in the distances between donors’ correspondence address and a static donor centre). Both the Mobile (platelet) and ‘No invitation’ participants showed characteristics distinct from other groups. The Mobile (platelet) group showed a markedly greater proportion of men and, from their donation history, were shown to be an especially committed group of donors. This group also had a lower proportion of participants who were less than 35 years old and of blood group O Rh(D) positive. In contrast, the ‘No invitation’ donors showed the highest proportion of participants in the 17–24 year age group, the highest proportion of new donors, the shortest average history of donation and the fewest donations in the 2 years prior to baseline.

**Table 5:** Demographic characteristics of the INTERVAL cohort by recruitment source^a^

Demographics	INTERVAL cohort				
*n* (%) or mean (SD)	Centre	Mobile (platelet)	Mobile (10-mile)	Mobile (30-mile)	No invitation
All donors	31,929 (100.0 %)	1741 (100.0 %)	5237 (100.0 %)	2409 (100.0 %)	7376 (100.0 %)
Sex					
Male	15,996 (50.1 %)	1379 (79.2 %)	2196 (41.9 %)	1110 (46.1 %)	3531 (47.9 %)
Female	15,933 (49.9 %)	362 (20.8 %)	3041 (58.1 %)	1299 (53.9 %)	3845 (52.1 %)
Age at baseline (years)	43.7 (14.4)	51.2 (13.2)	41.9 (11.7)	45.2 (13.6)	38.3 (14.2)
Age at baseline					
17–24	3866 (12.1 %)	82 (4.7 %)	544 (10.4 %)	268 (11.1 %)	1664 (22.6 %)
25–34	6688 (20.9 %)	166 (9.5 %)	1102 (21.0 %)	365 (15.2 %)	1915 (26.0 %)
35–44	5849 (18.3 %)	263 (15.1 %)	1205 (23.0 %)	430 (17.8 %)	1348 (18.3 %)
45–54	7233 (22.7 %)	446 (25.6 %)	1576 (30.1 %)	612 (25.4 %)	1291 (17.5 %)
55–64	5938 (18.6 %)	495 (28.4 %)	797 (15.2 %)	734 (30.5 %)	858 (11.6 %)
65+	2355 (7.4 %)	289 (16.6 %)	13 (0.2 %)	0 (0.0 %)	300 (4.1 %)
Ethnicity					
White	28,924 (90.6 %)	1644 (94.4 %)	4774 (91.2 %)	2227 (92.4 %)	6623 (89.8 %)
Asian	790 (2.5 %)	12 (0.7 %)	83 (1.6 %)	36 (1.5 %)	206 (2.8 %)
Black African	78 (0.2 %)	1 (0.1 %)	13 (0.2 %)	2 (0.1 %)	24 (0.3 %)
Black Caribbean	201 (0.6 %)	5 (0.3 %)	38 (0.7 %)	21 (0.9 %)	42 (0.6 %)
Black other	24 (0.1 %)	0 (0.0 %)	2 (0.0 %)	3 (0.1 %)	2 (0.0 %)
Chinese	120 (0.4 %)	1 (0.1 %)	12 (0.2 %)	4 (0.2 %)	27 (0.4 %)
Mixed	451 (1.4 %)	11 (0.6 %)	67 (1.3 %)	33 (1.4 %)	117 (1.6 %)
Other	94 (0.3 %)	2 (0.1 %)	14 (0.3 %)	4 (0.2 %)	24 (0.3 %)
Unknown	1247 (3.9 %)	65 (3.7 %)	234 (4.5 %)	79 (3.3 %)	311 (4.2 %)
Blood group					
A RhD positive (A+)	9414 (29.5 %)	635 (36.5 %)	1612 (30.8 %)	854 (35.5 %)	2222 (30.1 %)
A RhD negative (A-)	2405 (7.5 %)	209 (12.0 %)	371 (7.1 %)	207 (8.6 %)	552 (7.5 %)
B RhD positive (B+)	2783 (8.7 %)	121 (7.0 %)	436 (8.3 %)	258 (10.7 %)	646 (8.8 %)
B RhD negative (B-)	737 (2.3 %)	45 (2.6 %)	96 (1.8 %)	0 (0.0 %)	192 (2.6 %)
O RhD positive (O+)	11,589 (36.3 %)	485 (27.9 %)	1995 (38.1 %)	1073 (44.5 %)	2500 (33.9 %)
O RhD negative (O-)	3859 (12.1 %)	199 (11.4 %)	515 (9.8 %)	0 (0.0 %)	969 (13.1 %)
AB RhD positive (AB+)	880 (2.8 %)	40 (2.3 %)	164 (3.1 %)	0 (0.0 %)	209 (2.8 %)
AB RhD negative (AB-)	256 (0.8 %)	7 (0.4 %)	48 (0.9 %)	17 (0.7 %)	74 (1.0 %)
Unknown	6 (0.0 %)	0 (0.0 %)	0 (0.0 %)	0 (0.0 %)	12 (0.2 %)
Distance to nearest static donor centre (miles)					
0–4	21,298 (66.7 %)	570 (32.7 %)	3446 (65.8 %)	508 (21.1 %)	4583 (62.1 %)
5–9	5778 (18.1 %)	471 (27.1 %)	1329 (25.4 %)	818 (34.0 %)	1405 (19.0 %)
10–29	3946 (12.4 %)	649 (37.3 %)	377 (7.2 %)	1057 (43.9 %)	1174 (15.9 %)
30–59	538 (1.7 %)	36 (2.1 %)	37 (0.7 %)	13 (0.5 %)	151 (2.0 %)
60+	68 (0.2 %)	2 (0.1 %)	7 (0.1 %)	7 (0.3 %)	14 (0.2 %)
Unknown	301 (0.9 %)	13 (0.7 %)	41 (0.8 %)	6 (0.2 %)	49 (0.7 %)
Donor status at baseline					
New	453 (1.4 %)	0 (0.0 %)	6 (0.1 %)	8 (0.3 %)	925 (12.5 %)
Occasional	4180 (13.1 %)	35 (2.0 %)	1039 (19.8 %)	411 (17.1 %)	2247 (30.5 %)
More frequent	27,296 (85.5 %)	1706 (98.0 %)	4192 (80.0 %)	1990 (82.6 %)	4204 (57.0 %)
Donations (during the 2 years prior to baseline)					
All donations	3.47 (1.87)	4.28 (1.68)	3.06 (1.66)	3.35 (1.75)	2.39 (2.54)
Whole-blood donations	3.38 (1.73)	4.13 (1.51)	3.05 (1.65)	3.33 (1.73)	2.15 (1.92)
Other donations	0.08 (0.89)	0.15 (1.08)	0.01 (0.29)	0.02 (0.32)	0.23 (1.87)
Deferrals (during the 2 years prior to baseline)					
Deferral for low haemoglobin	2597 (8.1 %)	73 (4.2 %)	242 (4.6 %)	119 (4.9 %)	277 (3.8 %)
Any other deferral	10,174 (31.9 %)	461 (26.5 %)	1238 (23.6 %)	660 (27.4 %)	2095 (28.4 %)
Length of NHSBT donation history at baseline (years)	11.2 (8.4)	14.2 (8.4)	10.2 (8.1)	11.4 (9.1)	7.78 (7.71)

^a^For definitions see footnotes to Table 3

## Discussion

More than 45,000 blood donors have been successfully recruited into the NHSBT-embedded INTERVAL trial, making it the largest randomised study, worldwide, of the impact of more frequent donations on blood supplies and donor health. Unusually, our study had the ability to compare directly a range of participants’ characteristics between the INTERVAL trial and the target population. We found that there was broad similarity of participants in INTERVAL with the general blood donor population of England, notwithstanding some differences in age, sex and donation history. These observations lend support to the generalisability of the trial’s future results, at least for NHSBT. Nevertheless, any heterogeneity of the trial’s results according to the characteristics listed above will need to be studied to ensure its generalisability to the general donor population (Additional file 1). The current analysis also shows that a broad range of donors have participated in INTERVAL, enabling future assessment of the impact of increased donation frequency in relevant subgroups.

INTERVAL required a substantial commitment from donors: blood donations at regular (and more frequent than usual) intervals over a 2-year period and completion of online questionnaires every 6 months. Exclusion criteria were kept to a minimum, and all eligibility criteria were necessary for operational considerations (attendance at a static donor centre), ethics requirements (aged ≥18 years), scalability (access to email/Internet) and data integrity (completion of questionnaires at baseline). Recruitment was initially planned only from donors registered at a static donor centre. However, only about half of the donors from this source attended one of these centres during the recruitment period, rather than the approximately 70 % that would have been needed to meet recruitment targets. Given the shortfall in recruitment from the static donor centres, extra recruitment strategies were employed which focused on targeting groups of mobile session donors who were most likely to be able to transfer to a static donor centre to take part in the study and/or were of a significant size. The take-up rate in these groups of donors, while lower than that for the static centre donors, contributed almost 20 % of the total participants recruited into INTERVAL.

The trial’s participation rates varied according to the groups of donors targeted. Furthermore, donors recruited from different sources showed distinct characteristics; this was especially the case for the Mobile (platelet) and ‘No invitation’ participants. For the former group, this can probably be attributed to the commitment that is required to give platelet donations up to every 2 weeks and NHSBT’s platelet donor recruitment strategy.

We compared the characteristics of participants randomised into INTERVAL with the general donor population, focusing on features potentially relevant to trial outcomes. Although we found that the populations were generally similar, there were subtle differences in sex and age distributions. Moreover, a comparison of donation history indicated that the INTERVAL participants represented a longer-standing group of donors who had donated more frequently. It is possible that a more dedicated group of donors may make greater efforts (or have fewer other commitments) to attend more frequent donations. Hence, any impact observed in INTERVAL of inviting donors to give blood more frequently to increase blood supplies may be greater than that achieved in the general donor population. The data in this paper, however, suggest that the INTERVAL cohort were similarly resilient to iron deficiency following repeat donations as the general donor population, with deferral rates for low haemoglobin levels similar in both groups.

Donor centres offer permanent sites for blood donation, which are typically open daily during the working week in specific geographical locations across England. This is in contrast to temporary ‘mobile’ donation sessions in community venues, which visit locations at weekly or less frequent intervals. It is possible that the trial’s requirement to attend a static donor centre contributed, in part, to the subtle differences in characteristics of INTERVAL participants compared to the general donor population. In recent years, NHSBT’s strategy has been to increase the proportion of whole-blood donations collected in static donor centres; in 2015/2016 approximately 15 % of whole-blood donations were made at donor centres, and the target is to increase this to 25 % in 2020/2021. It is possible, therefore, that the INTERVAL cohort may be more representative of the general donor population in the future.

Not all donors who consented to participate in INTERVAL were randomised. Compared to the randomised group, consenting but non-randomised participants had a higher proportion of younger donors, a shorter history of donation, a smaller proportion of more frequent donors, a lower average number of donations and a higher proportion of non-white ethnic groups. Perhaps surprisingly, a greater proportion of randomised participants had a correspondence address that was 10 miles or more from a static donor centre.

## Conclusions

It is important that the conclusions from the analysis of RCT data can be appropriately generalised and, for this reason, RCTs should try to establish formally how representative the individuals recruited are of the target population. This is often not easy to achieve, but we had the opportunity to do this because of the existence of a national database. There was broad similarity of participants in INTERVAL with the general blood donor population of England, notwithstanding some differences in age, sex and donation history. Factors which differ between the recruited sample and the target population will need to be investigated as potential effect modifiers in the analysis of the trial data (using subgroup analyses or tests of interaction). However, it is not possible to rule out differences in unmeasured variables or unobservable characteristics, so there always remains an element of uncertainty in generalising the results of RCTs. Furthermore, we acknowledge that the extrapolation of INTERVAL’s results to blood services outside England and Wales is not straightforward, because blood services differ in several important respects, such as donor selection practices and policies about allowable inter-donation intervals.

## Additional file

Additional file 1: INTERVAL trial – statistical analysis plan for principal paper. (PDF 74 kb)

## Abbreviations

CI: Confidence interval
NHSBT: National Health Service Blood and Transplant
RCT: Randomised controlled trial
