# Supplementary material for: Recruitment and representativeness of blood donors in the INTERVAL randomised trial assessing varying inter-donation intervals
Source: Trials. 2016 Sep 20;17:458. doi: 10.1186/s13063-016-1579-7 (PMC5029021; doi:10.1186/s13063-016-1579-7)
Supplement: Additional file 1: — INTERVAL trial – statistical analysis plan for principal paper. (PDF 74 kb) [file 13063_2016_1579_MOESM1_ESM.pdf]

INTERVAL trial – Statistical analysis plan for principal paper

8 January 2016, final agreed version
Compiled by Simon Thompson, on behalf of the INTERVAL Trial Steering Committee

1. Aim

The purpose of this document is:

- to clarify the analyses to be conducted in the INTERVAL trial for the principal outcomes paper;
- to minimise misleading inferences that could arise from post hoc analyses.

This plan has been written in advance of looking at the 2-year outcome data from the trial, and is based on what was specified in the protocol [1].

The final version of this plan will be uploaded onto the INTERVAL trial website in advance of undertaking the principal trial analyses, and possibly published as an appendix to a relevant paper.

There will be a number of additional subsidiary papers from the INTERVAL trial and its sub-studies, which are not discussed in detail here.

2. Summary of trial design

Consenting blood donors in 25 centres are randomised to different intervals between blood donations: men to 8w or 10w vs. the standard 12w, and women to 12w or 14w vs. the standard 16w. Around 45,000 blood donors have been randomised, resulting in about 7500 in each of these six groups. Randomisation is stratified by sex and centre, and minimized according to age, weight and donor status (new vs. existing). Planned follow-up is for 2 years.

Trial blood samples are collected at baseline and at 2 years, together with information on health outcomes and symptoms from online questionnaires; this includes the SF36 quality of life scale from which physical and mental component scores can be calculated. Similar online questionnaires are also administered at 6-month intervals during the trial; these include the SF12 quality of life scale from which physical and mental component scores can also be calculated. All the questionnaires contain questions about potential side-effects of blood donation. Haemoglobin and a large range of other haematology variables are measured by a Sysmex analyser on the trial blood samples from the baseline and 2-year visits. Cognitive function, recent physical activity and pica (eating non-food items) are assessed by questionnaire at 2 years.

The primary outcome of the trial is:

- the amount of blood collected over the 2-year period.

The key secondary outcome is:

- the physical component score (PCS) of the SF36 at 2 years.

In terms of the amount of missing data:

- The data on blood collected are complete by design.
- The SF36 data at 2 years are incomplete since some donors have withdrawn from the trial before 2 years, or fail to complete the 2-year questionnaire despite being sent reminders.
- The baseline questionnaire data are complete, since donors are not randomised until this has been done; nonetheless a small number of donors did not fully complete the SF36 at baseline.
- The 6-monthly and 2-year questionnaires are not available for donors who have withdrawn from the trial before these were due, or who failed to complete them despite being sent reminders.
- A small number of baseline trial blood samples are not available, due to inadequate venepuncture, transport or other problems.
- A larger number of 2-year trial blood samples are not available, because some donors did not attend for donation before or shortly after their 2-year anniversary of randomisation.

The trial was powered on the basis of being able to detect clinically relevant differences between randomised groups (a power of at least 80% for a 5% difference in amount of blood collected and a 3% difference in the SF36 physical component score), in subgroups of donors that include at least 20% of the men or 20% of the women randomised.

3. Timelines and scope

Recruitment to the trial was from June 2012 to June 2014. The final 2-year follow-up blood samples and questionnaires are scheduled to be obtained in July 2016. Dummy statistical analyses (<u>blinded</u> to randomised group) will be run from January 2016, according to the plan outlined here, in order to develop analysis code and streamline the final analyses. The main trial data will be analysed from August 2016 and results presented to the Trial Steering Committee, with the intention of submitting a paper for publication before the end of 2016. Presentations at relevant conferences, and dissemination to NHSBT, are also planned.

The trial now includes a number of sub-studies and add-ons. The results from these sub-studies will <u>not</u> form part of the principal INTERVAL trial paper. The main such sub-studies are:

(i) Data on donor attitudes collected by online questionnaire at 2 years.
(ii) Detailed measures of iron status at baseline and 2 years (e.g. ferritin, transferrin).
(iii) Health economic evaluation of within-trial and out-of-trial options for NHSBT policies on blood donation.
(iv) Phase II trial: In those participants who consent, the continuation of allocated donation intervals for up to 4 years, and their further randomisation to more intensive INTERVAL Study Administration Team (ISAT) vs. standard NHSBT reminders to attend. About 50% of donors in the main trial have agreed to take part in Phase II.

(v) Collection of physical activity data from wrist-worn monitors just after 2 years in a subset of about 6000 donors from across all six randomised groups who consent to take part.

(vi) Phase III trial: Serial research blood samples taken over 2 or more donations during October 2015 to June 2016, in about 3600 donors who consent to take part.

4. Overall analysis strategy

The trial will be reported according to CONSORT guidelines. The analyses will include all donors randomised, and consider them in the groups as randomised (i.e. intention to treat). The very small number of donors who have withdrawn consent for their data to be used will necessarily be omitted.

Men and women will always be analysed separately.

For the primary outcome and key secondary outcome (see Section 6), there are two principal comparisons for men: 8w vs. 12w, and 10w vs. 12w. Similarly there are two principal comparisons for women: 12w vs. 16w, and 14w vs. 16w. The analysis will be performed by including all three randomised groups for each sex, and making the two relevant contrasts of groups. Comparisons of 8w vs. 10w groups for men, and of 12w vs. 14w groups for women, will be informal (i.e. from a graph of the results).

For other secondary outcomes (see Section 7) and for assessing interactions (see Section 10), in order to reduce the number of statistical tests performed, analyses will assess the linear trend across randomised groups for each sex separately (i.e. 8w, 10w, 12w for men; 12w, 14w, 16w for women).

Analyses will be presented first unadjusted, and then adjusted for baseline covariates (see Section 9).

To guard against false-positive results given the multiple comparisons being performed, stringent levels of statistical significance will be required before strong evidence of differences between randomised groups can be claimed (see Section 11).

The main analyses will omit donors with missing data on the relevant outcome (i.e. a complete-case analysis). Secondary analyses will be undertaken to address the potential biases caused by missing data (see Section 12).

5. Descriptive analyses

The trial protocol paper has been published [1], and it is anticipated that the recruitment to the trial will have been described in further detail in a separate paper, to which reference can be made. So the principal outcomes paper can focus on:

- Baseline characteristics by randomised group, for example: age, past donation history (new vs. existing donor, number of donations over last 2 years, low haemoglobin deferral rate over the last 5 years), SF36 physical and mental health

component scores, weight, body mass index (BMI), haemoglobin. Results will be expressed as mean (SD) or number (%). The amount of missing data will be shown. No statistical testing of differences between groups will be undertaken.

- Flow of donors through the trial after randomisation, as a CONSORT diagram, including withdrawals and completeness of the outcomes data.
- Adherence to allocated intervals over the 2-year period by randomised group, presented as Kaplan-Meier plots of time between attendances. Those who do not re-attend after a given donation until after the 2-year anniversary of randomisation will be censored at 2 years.

6. Primary outcome and key secondary outcome

The primary outcome is:

- The amount of blood (i.e. the number of units of blood) collected over the 2-year period, defined from the day of randomisation to (and not including) the exact 2-year anniversary of this date.

The key secondary outcome is:

- The physical component score (PCS) of the SF36 at 2 years. This questionnaire was administered online and sent to donors on their 2-year anniversary. If the questionnaire was not completed by the 2-year anniversary plus 30 days, despite being sent reminders, the outcome will be considered missing. The PCS is calculated from individual questions according to the SF36 manual [2], and allows for a small number of answers to be missing.

These results will be presented as the mean (SD and 95%CI) in each randomised group, and mean difference (95%CI) from the standard interval group for each sex.

7. Other secondary outcomes

The statistical analysis of other secondary outcomes will assess the linear trend across randomised groups within each sex, using linear regression for continuous outcomes and logistic regression for binary outcomes. The following secondary outcomes will be included in the principal trial paper:

- Deferrals for low haemoglobin, and deferrals only for other reasons, over 2 years in each randomised group. These will be presented as percentage (95%CI) per donation session attended.
- Haemoglobin at 2 years, presented as mean (95%CI) in each randomised group, and percentage (95%CI) with haemoglobin <13.5 g/dL for men or <12.5 g/dL for women.
- SF36 mental component score at 2 years, presented as mean (95%CI) in each randomised group.
- Cognitive function at 2 years: five tests assessing attention, reaction time, executive function, episodic memory, and fluid intelligence. These will be presented as mean (95%CI) in each randomised group.

- Recent physical activity questionnaire (RPAQ) at 2 years: overall score, presented as mean (95%CI) in each randomised group. This score may be calibrated against the activity monitor data available in a subset of participants.
- Any adverse event over 2 years from the 6-monthly and 2-year questionnaires: any reporting of visiting doctor or hospital (which was subsequently confirmed) for heart failure, heart attack, angina, stroke, transient ischaemic attack, falls, or transport accidents. This will be presented as the percentage (95%CI) of participants in each randomised group.
- Any fainting at a donation session over 2 years in each randomised group, presented as percentage (95%CI) per donation session attended.
- Reported symptoms over 2 years from the 6-monthly and 2-year questionnaires: greater tiredness than usual, greater breathlessness than usual, palpitations, dizziness, feeling faint or fainting, chest pain, restless leg syndrome. Each symptom will be presented as the percentage (95%CI) of participants in each randomised group.
- Pica reported on the 2-year questionnaire, presented as the percentage (95%CI) of participants in each randomised group.

Secondary outcomes above that refer to "over 2 years" combine data from multiple donations attended, or multiple questionnaires answered, by each individual. Allowance for the consequent extra uncertainty due to this clustering by individual will be addressed by using robust SEs.

8. Descriptive analyses of outcomes

Some additional data may be presented, probably graphically, but without statistical testing. These include:

- Response rates to the 6-monthly and 24-month questionnaires by randomised group, and the time period since randomisation over which the questionnaires were returned.
- Availability of trial blood samples at 2 years by randomised group, and the time period since randomisation over which they were obtained. These blood samples were collected at the last donation visit before the 2-year anniversary of randomisation, or subsequently if the donor did not attend. A time period up to 120 days after the 2-year anniversary or 31 July 2016 (whichever is sooner) is permitted.
- The trajectory of SF36 and SF12 physical component scores over 2 years by randomised group.
- A comparison of low haemoglobin deferral rates (per donation session attended) in the first and second year after randomisation by randomised group.
- The separate items making up the composite secondary outcome of any adverse event (Section 7), and the number of recorded deaths, by randomised group.

Also, additional analyses may be undertaken to help fully understand the results presented in the paper. These are likely to include:

- Rates of deferral and of fainting expressed per person and over time, rather than per donation session attended.
- Symptoms and adverse events reported in each 6-month questionnaire.
- Results by recruitment source (as defined in the INTERVAL recruitment paper), especially adherence and withdrawal rates.
- Investigation of other determinants of adherence.
- Correlation between outcomes, especially reported symptoms.

9. Baseline covariate adjustment

Differences between groups, or trends across groups, will be adjusted for baseline characteristics, by analysis of covariance for continuous outcomes and by logistic regression for binary outcomes. Baseline characteristics will include the baseline version of the outcome (where relevant), centre (as a random effect), age, weight, and new vs. existing donor. Analyses will also adjust for season of ascertainment (quarters of the year) for outcomes assessed at 2 years. If the baseline data are substantially incomplete, a missing indicator method will be used so that all data can be included [3].

10. Subgroups

Subgroup analyses will be carried out only for the primary outcome and the key secondary outcome, assessing the relevant statistical interaction with baseline characteristics by linear regression. All three randomised groups for each sex will be included, and the interaction test will assess whether the linear trend across them varies according to the baseline characteristic considered. Continuous baseline characteristics will be analysed as linear terms in the regression models, but results presented in groups as specified below. The subgroups to be compared will be:

- According to age: linear in regression models, but presented as <50 vs. 50+ years.
- According to weight: linear in regression models, but presented as <70 vs. 70+ kg for men and <60 vs. 60+ kg for women.
- According to donation history (number of donations in 2 years previous to randomisation): linear in regression models, but presented as below / above the sex-specific median.
- According to genotype: carriers vs. non-carriers of human haemochromatosis (HFE) gene variants (i.e. C282Y and/or H63D variants, which have an anticipated combined prevalence of over 30% [4]).
- According to haemoglobin: linear in regression models, but presented as below / above the sex-specific median.

11. Controlling false positive rates

The P-values presented will not be adjusted for multiplicity, but interpretation needs to take into account the multiple statistical tests that have been performed.

- There are two main outcomes (see Section 6: amount of blood collected and PCS at 2 years), two sexes, and two main comparisons for each sex (e.g. 10w vs. 12w and

8w vs. 12w for men, see Section 5). This yields eight statistical tests, which suggests considering only P<0.005 (i.e. roughly 0.05/8) as providing strong evidence of differences in means between groups.

- There are 20 other secondary outcomes (see Section 7: two types of deferral, haemoglobin, SF36 mental component score, five cognitive function tests, RPAQ, any adverse event, fainting at donation session, seven symptoms, and pica), and two sexes. This yields 40 statistical tests, which suggests considering only P<0.0002 (i.e. roughly 0.01/40) as providing strong evidence of trends across groups.
- For the tests of interaction, there are two outcomes analysed, two sexes, and five baseline characteristics (see Section 10). This yields 20 statistical tests, which suggests considering only P<0.0005 (i.e. 0.01/20), as providing strong evidence of differences in trends between subgroups.

This strategy controls the overall false positive rate to under 0.07 (i.e. 0.05 for the primary outcome and key secondary outcome, 0.01 for the other secondary outcomes, and 0.01 for the interactions). Interpretation of results will also take account of internal consistency across outcomes, as well as clinical plausibility based on prior evidence.

12. Missing data

Missing data for the SF36 physical component score (PCS) at 2 years due to non-completion of the online questionnaire will be the most important missingness issue. Because PCS can also be obtained from the 6-monthly SF-12 questionnaires comparable to SF-36, we will jointly model PCS derived from all questionnaires completed by a participant during follow-up to better account for missingness. Each participant can have up to a maximum of five PCS assessments (i.e. at baseline, 6 months, 12 months, 18 months, and 24 months – termed questionnaire number 1 – 5 below) and correlation is to be expected in the participant responses. For each sex separately, we will fit a linear mixed model with PCS as the dependent variable and fixed effects as randomised group, questionnaire number, the interaction of randomised group with questionnaire number, and other baseline covariates (see Section 9); random intercepts for centre and participant will be included to model between-centre differences and within-subject correlations. The effects of randomised group (2 df), questionnaire number (4 df), and their interaction (8 df) will be coded as dummy variables to ensure the most general model of mean longitudinal trajectories. The mixed model will be used to obtain marginal means of PCS by randomised group and questionnaire number, from which primary inferences will concern differences in the 24-month PCS marginal means. The model may be extended to include (i) other baseline covariates, (ii) deferral at previous blood collections, (iii) number of blood collections made, and (iv) the number of reminders at 2 years before a response was obtained amongst responders [5].

Missing data in other secondary outcomes will also occur, in particular for 2-year haemoglobin, the 2-year questionnaire data (SF36 mental component score, cognitive outcomes, RPAQ, pica), and the 6-monthly and 24-month questionnaires in which adverse

events and symptoms are reported. A similar scheme of joint modelling can be used for these outcomes if thought necessary.

13. Proposed tables and figures

The above analyses would lead to the following tables and figures in the paper. Some could be supplementary material.

- Table 1: Baseline characteristics by randomised group
- Figure 1: CONSORT diagram, including reasons for withdrawals
- Figure 2: Adherence to specified donation intervals
- Table 2: Primary outcome and key secondary outcome, unadjusted and adjusted
- Figure 3: Primary outcome and key secondary outcome
- Table 3: Other secondary outcomes, unadjusted and adjusted (grouped by 'system', such as cardiovascular / neurological / other)
- Figure 4: Other secondary outcomes
- Figure(s): Descriptive outcomes
- Figure(s): Subgroup findings for primary outcome and key secondary outcome

14. References

1. Moore C et al. The INTERVAL trial to determine whether intervals between blood donations can be safely and acceptably decreased to optimise blood supply: study protocol for a randomised controlled trial. *Trials* 2014, **15**: 363.

2. Maruish ME (ed.). *User's Manual for the SF-36v2 Health Survey (3rd ed.)*. Lincoln, RI: Quality Metric Incorporated, 2011.

3. White IR, Thompson SG. Adjusting for partially missing baseline measurements in randomised trials. *Statistics in Medicine* 2005; **24**: 993-1007.

4. Merryweather-Clarke AT, Pointon JJ, Shearman JD, et al. Global prevalence of putative haemochromatosis mutations. *J Med Genet* 1997; **34**: 275-278.

5. Wood AM, White IR, Hotopf M. Using number of failed contact attempts to adjust for non-ignorable non-response. *Journal of the Royal Statistical Society (A)* 2006; **169**: 525–542.
